# Very Late-Onset Lingual Necrosis Post-radiation in an Adult: A Case Report

**DOI:** 10.7759/cureus.89828

**Published:** 2025-08-11

**Authors:** Lina Benchillal, Layal Abi-Khalil, Didier Dequanter, Lamia El Ouahabi, Gaëtan Cavelier

**Affiliations:** 1 Department of Otorhinolaryngology and Head and Neck Surgery, Centre Hospitalier Universitaire (CHU) Saint-Pierre, Brussels, BEL; 2 Department of General Surgery, Centre Hospitalier Universitaire (CHU) Saint-Pierre, Brussels, BEL

**Keywords:** head and neck cancer, late radiation side-effect, lingual artery occlusion, lingual necrosis, oral cancer, radiotherapy complications, tongue necrosis

## Abstract

Lingual necrosis is a rare but serious late complication of radiotherapy for head and neck cancer, despite the tongue's rich vascular supply. We report the case of a 59-year-old man who developed unilateral tongue necrosis 16 months after chemoradiotherapy for oropharyngeal carcinoma. A cervico-facial CT scan showed occlusion of the right lingual artery and compensatory dilation of collateral vessels. Surgical debridement of necrotic tissue was performed, and histopathology confirmed the lingual necrosis. The delayed onset and extent of necrosis in this case suggest progressive, radiation-induced microvascular injury. This case highlights the importance of prolonged follow-up and early recognition of atypical post-treatment lesions.

## Introduction

Radiotherapy is part of the therapeutic arsenal in the treatment of head and neck cancers. Despite its clinical efficacy, this treatment is also associated with described but less frequent complications, such as lingual necrosis. The mobile tongue, although richly vascularized by the lingual arteries, can suffer from severe hypoperfusion due to vascular damage after radiotherapy, leading to intimal hyperplasia, medial necrosis, and secondary fibrosis [1]. This physiopathological process will subsequently lead to progressive tissue necrosis [2-4]. Although giant cell arteritis is classically reported as a primary cause of lingual necrosis, this association is rare and primarily described in isolated case reports [4,5]. The differential diagnosis remains broad and includes embolic or thrombotic events, severe atherosclerotic carotid stenosis, systemic vasculitis, coagulopathies, infections, vasopressor-induced ischemia, local trauma, and tumor recurrence [3-13]. Establishing an accurate diagnosis is therefore complex and requires a careful evaluation of clinical, radiological, and histopathological findings [6,10].

Lingual necrosis due to radiotherapy, although rare, can be debilitating and manifest itself in intense pain, persistent ulceration, or even bone exposure in advanced forms [6]. Clinical diagnosis is challenging and relies on a combination of various clinical and radiological factors. Its diagnosis is based on a set of clinical and radiological arguments and requires a rigorous differential diagnosis to eliminate any tumor recurrence.

In this context, we report a clinical case of lingual necrosis that occurred more than a year after radio-chemotherapy treatment for a lingual squamous cell carcinoma, thus illustrating the diagnostic and therapeutic challenges associated with this rare complication. This case highlights the importance of prolonged monitoring of patients treated with radiotherapy as well as the necessity of early management in the face of any atypical post-therapeutic lesion.

## Case presentation

A 59-year-old man was treated with concomitant radio-chemotherapy (cisplatin 100 mg/m² every three weeks, 70 Gy to the right oropharyngeal primary tumor and its lymph node extensions in bilateral II/III areas and right V; 56 Gy prophylactic to the Ib-V bilateral lymph node zone, VI and rhinopharyngeal bilateral) for a non-keratinizing invasive squamous cell carcinoma of the right oropharynx staged cT4aN2bM0 p16 negative in November 2023.

In addition, the patient's clinical history reveals high blood pressure treated with amlodipine 10 mg and perindopril 10 mg in the context of active smoking with a Brinkman index of 120 and no alcohol consumption. Moreover, the patient presented with polytoxicomania after consuming cannabis, cocaine, and heroin for 10 years, stopped four years ago, and is currently being substituted with methadone. There was no clinically significant bleeding, no documented thrombocytopenia, or history of thromboembolic events.

**Figure 1:**
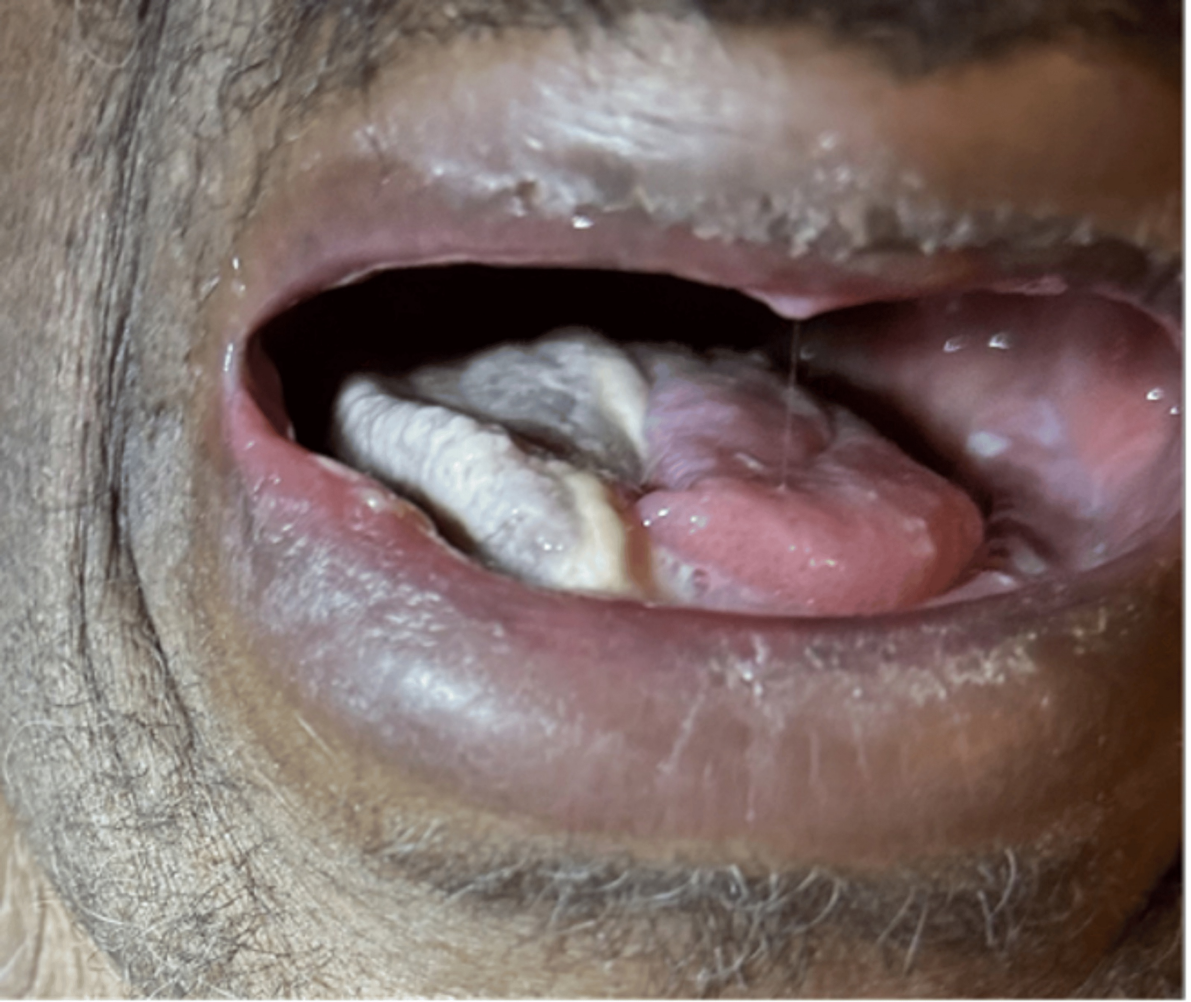
Trismus, limiting mouth opening, accompanied by extensive necrosis affecting the right half of the tongue, with associated edema and discoloration compromising speech and swallowing functions

**Figure 2:**
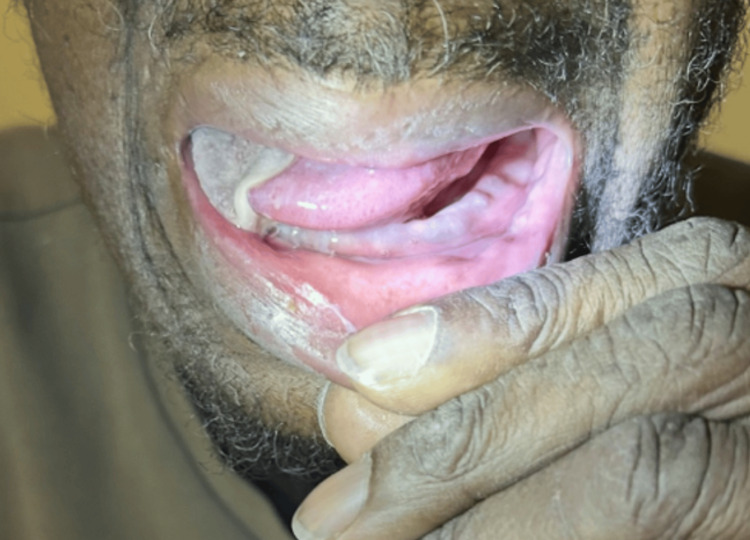
Right-lateralized lingual mobility observed during examination, indicating asymmetrical tongue movement with deviation toward the affected side

In February 2025, the patient sought consultation for glossodynia and aphagia, which were associated with involuntary weight loss resulting in a BMI of 19.2 kg/m². Additionally, there was no fever, immunosuppression, or infectious context. The ear-nose and throat clinical examination revealed trismus limiting mouth opening as well as necrosis affecting the right half of the tongue, without affecting the base of the tongue, with a disorder of right lateralized lingual mobility (Figures 1, 2).

**Figure 3:**
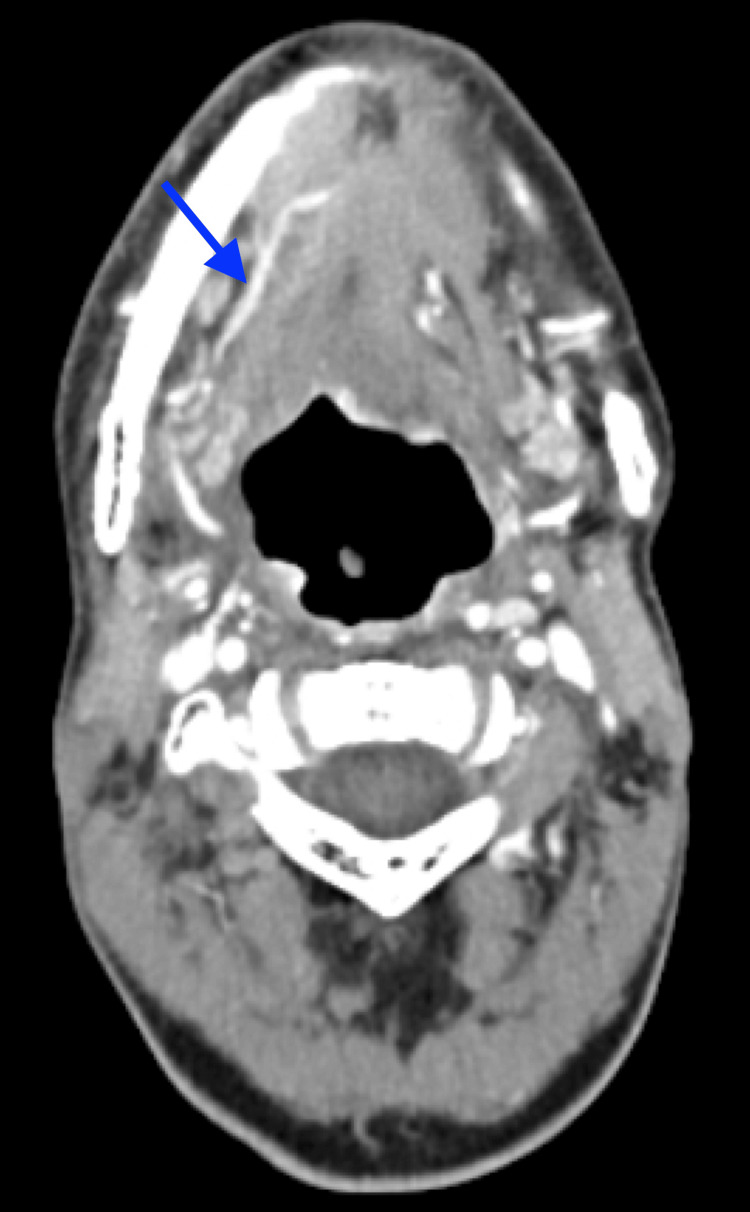
A computed tomography (CT) scan showing opacification of the right lingual artery with dilation of the supplementary arteries without signs of tumor recurrence

The general blood test was within normal limits. A cervico-facial computed tomography (CT) scan was then performed and showed opacification of the right lingual artery with dilation of the supplementary arteries without signs of tumor recurrence (Figure 3).

**Figure 4:**
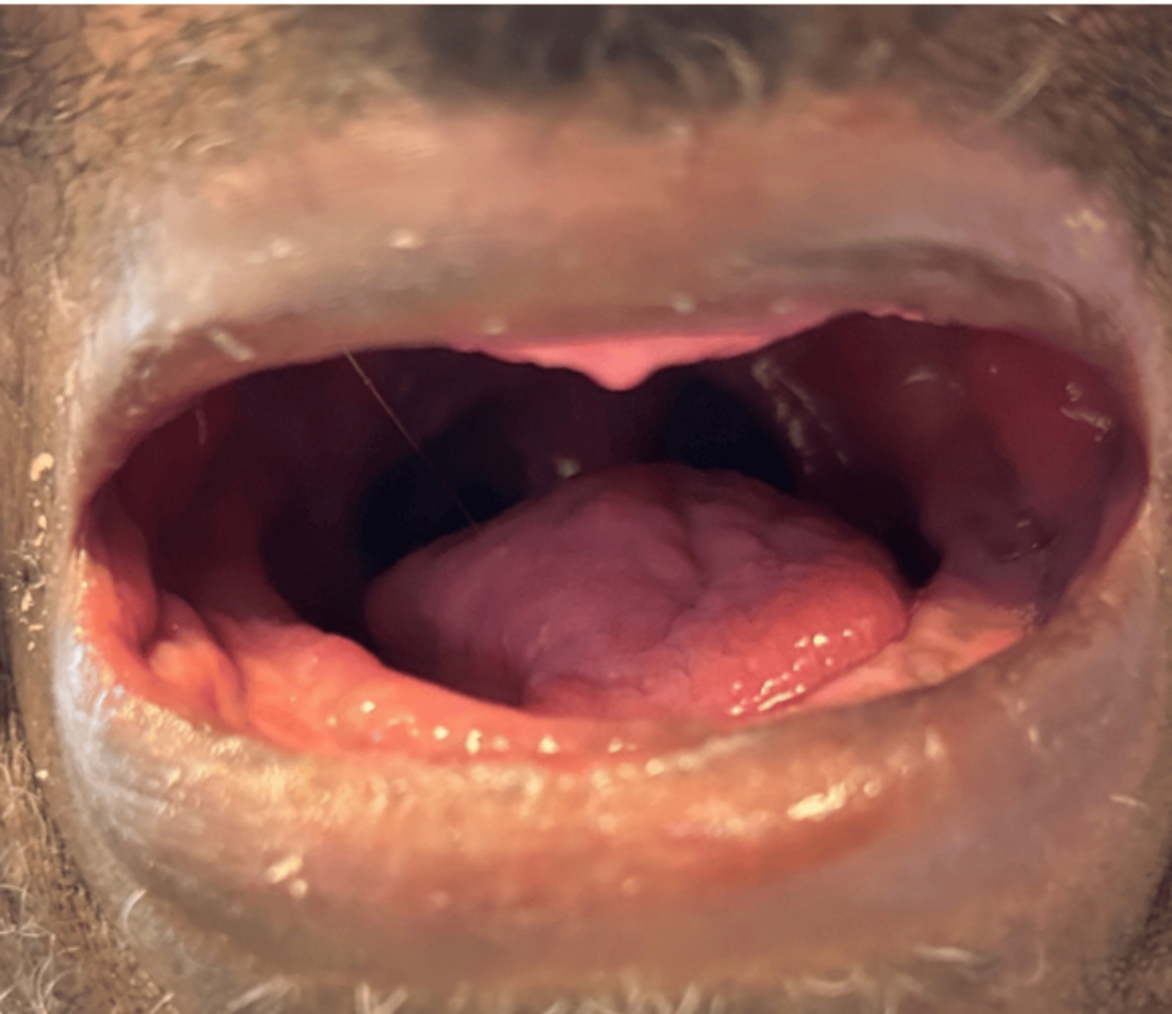
Intra-oral post-operative view following right hemiglossectomy, demonstrating satisfactory wound healing and no signs of local complications

The necrotic lingual tissue was surgically debrided (Figure 4), and numerous biopsies, through panendoscopy, were performed. Subsequently, histopathological analysis supported the diagnosis of post-radiation fibrosis, with no evidence of malignancy found in either the necrotic lingual tissue or in an additional biopsy taken from the right glossotonsillar sulcus. Following surgery, a nasogastric feeding tube was placed for three days, after which the patient resumed an oral liquid diet.

During the ongoing follow-up, the patient has been regularly evaluated in ENT, dietary, and speech therapy consultations. He currently maintains a soft-texture and enriched liquid diet. A control MRI and CT scan performed in April 2025 revealed no significant changes or suspicious lesions.

## Discussion

Lingual necrosis is rarely observed, due to the vascularization of the tongue, ensured by the lingual, facial, and ascending pharyngeal arteries and their numerous anastomoses [2,3,14]. This rich vascular network protects the mobile tongue from ischemic events, making any necrotic phenomenon particularly unusual.

In the literature, reported cases of lingual necrosis are most often unilateral and predominantly affect the anterior third of the tongue. The base of the tongue is, on the other hand, exceptionally affected [15]. Our case illustrates that this type of necrosis can affect an entire hemilingual region and also that this phenomenon can be observed at a distance from the initial radiotherapy treatment.

As part of the differential diagnosis of lingual necrosis, several etiologies were considered and subsequently ruled out in our patient. Giant cell arteritis was deemed unlikely due to the absence of suggestive clinical signs [2,16]. An embolic or thrombotic origin was excluded, given the localized and progressive nature of the lesion in a patient with no cardioembolic risk factors, as well as the subacute evolution over several weeks. The cervico-facial CT scan, which revealed only occlusion of the right lingual artery with collateral circulation, allowed us to rule out significant carotid artery stenosis. The hypothesis of systemic vasculitis could not be fully explored in the absence of specific immunological marker assays, although no suggestive signs were found. Given the clinical picture, this etiology remains unlikely. The hypothesis of coagulopathies was ruled out due to the absence of clinically significant bleeding, documented thrombocytopenia, or a history of thromboembolism. There was no fever, immunosuppression, or infectious context to suggest an invasive infectious origin. As the patient had neither been in intensive care nor received vasopressors, ischemic origin secondary to this type of treatment was not considered. No mechanical trauma or local compression factors were identified. Finally, the distinction between lingual necrosis and tumor recurrence can be clinically and radiologically difficult. In our patient, radiological assessment excluded recurrence.

In the absence of other causes, the diagnosis of post-radiation necrosis was retained despite the time taken for the onset of this rare complication and its extension. Our patient was treated with concomitant chemoradiotherapy for a non-keratinizing invasive squamous cell carcinoma of the right oropharynx.

Regarding radiotherapy, late effects are defined by the Radiation Therapy Oncology Group (RTOG) as those appearing more than 90 days after the start of radiotherapy treatment [17]. Among the most frequently described toxicities are xerostomia, dysphagia, caries, osteoradionecrosis (often mandibular), burning mouth syndrome, trismus, pharyngoesophageal strictures, skin lesions, and endocrinopathies such as hypothyroidism or hyperparathyroidism [11-13].

Among the late complications, post-radiation lingual necrosis remains a rare exception. In oropharyngeal cancers, the main risk factors for post-radiotherapy soft tissue necrosis include a depth of tumor invasion > 1.2-1.4 cm, tumor size > 2.5 cm, fraction dose > 2.3 Gy to the tumor bed, and the presence of grade 3 acute mucositis [17]. Few cases have been reported in the literature, as described by Aguiar et al. [2] and Curi et al. [15]. However, none of these cases describes such a complication with such a delay and such an extension in a young patient. In our case, the necrotic phenomenon could be explained by a progressive and irreversible impairment of the local microvascularization and a homolateral lingual artery occlusion [2,15].

The pathophysiology is based on Marx's "3H" model, which comprises hypovascularization, hypocellularity (due to cell death), and tissue hypoxia [18]. These phenomena are exacerbated by radiation-induced fibrosis, which further impairs the regeneration of normal tissues [18]. A histopathological study revealed seven typical lesions of irradiated tissues: hyperemia, endarteritis, thrombosis, cell loss, hypovascularization, medullary fat infiltration, and fibrosis. Early lesions, such as hyperemia and cell death, occur within the first six months; later lesions, like fibrosis and thrombosis, appear more gradually [4,15].

In our case, the unilateral necrosis, associated with its delayed occurrence 16 months after (chemo)radiotherapy, and the absence of tumor recurrence or any other cause, strongly suggest that the necrosis is secondary to the vascular and tissue effects of irradiation.

The initial treatment includes symptomatic management, which combines analgesics, local care, and antibiotics in cases of superinfection. Hyperbaric oxygen therapy may be offered in certain cases, although its effectiveness remains debated. Surgical debridement may sometimes be necessary [15].

This case highlights the importance of prolonged and multidisciplinary follow-up for patients treated for head and neck cancer and sheds light on a rare but potentially severe complication of a treatment that is now widely used. The patient remains under ongoing follow-up in our clinic.

## Conclusions

Post-radiotherapy lingual necrosis constitutes a rare but severe complication, the diagnosis of which can be complex, particularly in an oncological context. Our observation illustrates the late radiation side effects, as well as the therapeutic challenges they raise. The medical history of our patient highlights the importance of rigorous and prolonged clinical follow-up for patients treated for head and neck carcinoma, particularly in the presence of any atypical lesion. Early recognition of this complication allows for appropriate management, thereby limiting functional sequelae and improving the quality of life for patients.
